# Genomic comparison and phenotypic profiling of small colony variants of *Burkholderia pseudomallei*

**DOI:** 10.1371/journal.pone.0261382

**Published:** 2021-12-15

**Authors:** Noorfatin Jihan Zulkefli, Cindy Shuan Ju Teh, Vanitha Mariappan, Soo Tein Ngoi, Jamuna Vadivelu, Sasheela Ponnampalavanar, Lay Ching Chai, Chun Wie Chong, Ivan Kok Seng Yap, Kumutha Malar Vellasamy

**Affiliations:** 1 Faculty of Medicine, Department of Medical Microbiology, Universiti Malaya, Kuala Lumpur, Malaysia; 2 Faculty of Health Sciences, Centre of Toxicology and Health Risk Studies (CORE), Universiti Kebangsaan Malaysia, Kuala Lumpur, Malaysia; 3 Faculty of Medicine, Department of Medicine, Universiti Malaya, Kuala Lumpur, Malaysia; 4 Faculty of Science, Institute of Biological Sciences, Universiti Malaya, Kuala Lumpur, Malaysia; 5 School of Pharmacy, Monash University Malaysia, Bandar Sunway, Selangor, Malaysia; 6 Institute for Research, Development & Innovation, International Medical University, Kuala Lumpur, Malaysia; 7 Sarawak Research and Development Council, Kuching, Sarawak, Malaysia; University of Toledo College of Medicine and Life Sciences, UNITED STATES

## Abstract

*Burkholderia pseudomallei* (*B*. *pseudomallei*) is an intracellular pathogen that causes melioidosis, a life-threatening infection in humans. The bacterium is able to form small colony variants (SCVs) as part of the adaptive features in response to environmental stress. In this study, we characterize the genomic characteristics, antimicrobial resistance (AMR), and metabolic phenotypes of *B*. *pseudomallei* SCV and wild type (WT) strains. Whole-genome sequence analysis was performed to characterize the genomic features of two SCVs (CS and OS) and their respective parental WT strains (CB and OB). Phylogenetic relationship between the four draft genomes in this study and 19 publicly available genomes from various countries was determined. The four draft genomes showed a close phylogenetic relationship with other genomes from Southeast Asia. Broth microdilution and phenotype microarray were conducted to determine the AMR profiles and metabolic features (carbon utilization, osmolytes sensitivity, and pH conditions) of all strains. The SCV strains exhibited identical AMR phenotype with their parental WT strains. A limited number of AMR-conferring genes were identified in the *B*. *pseudomallei* genomes. The SCVs and their respective parental WT strains generally shared similar carbon-utilization profiles, except for D,L-carnitine (CS), g-hydroxybutyric acid (OS), and succinamic acid (OS) which were utilized by the SCVs only. No difference was observed in the osmolytes sensitivity of all strains. In comparison, WT strains were more resistant to alkaline condition, while SCVs showed variable growth responses at higher acidity. Overall, the genomes of the colony morphology variants of *B*. *pseudomallei* were largely identical, and the phenotypic variations observed among the different morphotypes were strain-specific.

## Introduction

Intracellular bacterial pathogens are adaptable to various conditions and environmental stress in host cells. However, due to population heterogeneity [1], only small subsets of bacterial populations are able to endure harsh conditions, such as antibiotic stress, pH fluctuation, osmotic pressure, and nutrient-depauperate environment. Formation of small colony variants (SCVs) is one of the important bacterial survival strategies, particularly in intracellular bacteria [2]. SCV is characterized by the pinpoint colony, slow growth rate, and rapid reversion to wild type (WT) phenotype [3]. In general, SCVs have been associated with chronic, relapse and persistent infections [4]. Among the intracellular pathogens known to produce SCVs are *Staphylococcus aureus* [5], *Neisseria gonorrhoeae* [6], *Burkholderia cepacia* [7], and *Burkholderia pseudomallei* [8].

*B*. *pseudomallei*, a Gram-negative bacteria found in soil and contaminated water, is the causative agent of melioidosis, a life-threatening infection endemic mainly in Southeast Asia and Australia [9]. Clinical presentations of melioidosis are diverse; ranging from benign skin infection to fulminating chronic infection, and the organs typically involved are lungs, spleen, liver, and prostate [10,11]. Due to the severe infection, aerosol infectivity, and intrinsic resistance to a broad range of antibiotics, *B*. *pseudomallei* has been classified as a Tier 1 biological agent by the U.S. Federal Select Agent Program, presenting the greatest risk of deliberate misuse and posing a severe threat to the public health and safety [12].

Previous studies on *B*. *pseudomallei* SCVs had reported higher antimicrobial resistance (AMR) [8], an increase in biofilm production [13], a decrease in virulence [14], and an increase in persistence as compared to the WT strains [15]. Despite the implication of the transmission and treatment of *B*. *pseudomallei* infection, there is a lack of understanding of the genotypic and phenotypic characteristics of *B*. *pseudomallei* SCVs. Thus, the objective of this study was to compare the *B*. *pseudomallei* SCVs and WTs based on the genomic and phenotypic characteristics. The phylogenetic relationship of SCVs and WTs were also compared to other publicly available *B*. *pseudomallei* genomes from different countries.

## Materials and methods

### Bacterial strains and culture conditions

A total of four *B*. *pseudomallei* strains (CB, CS, OB, and OS) analyzed in this study were retrieved from the archival collection of the Department of Medical Microbiology, Universiti Malaya. The strains consisted of two WTs (CB and OB) and their corresponding SCVs (CS and OS, respectively). The strains were isolated from the blood of two separate melioidosis cases in University Malaya Medical Centre (UMMC), Malaysia, and two different morphotypes (WT and SCV) were found as previously described by Ramli *et al*. [13]. In brief, these four strains, regardless of their morphotypes, were previously identified as sequence type (ST) 46 according to multilocus sequence typing (MLST) analysis [16]. Based on pulsed-field gel electrophoresis (PFGE) analysis of the colonial variants, CB (WT) and CS (SCV) were 95% similar, but OB and OS shared only 67% similarity [16]. All of the strains were revived on nutrient agar at 37°C for 24 hours and 48 hours for WTs and SCVs, respectively. The strains were then sub-cultured three times on nutrient agar to confirm homogeneity. The SCV phenotype was stable (no reversion to WT phenotype) as confirmed by observation of the colony morphology after sub-culturing.

### Experimental design

**Fig 1:**
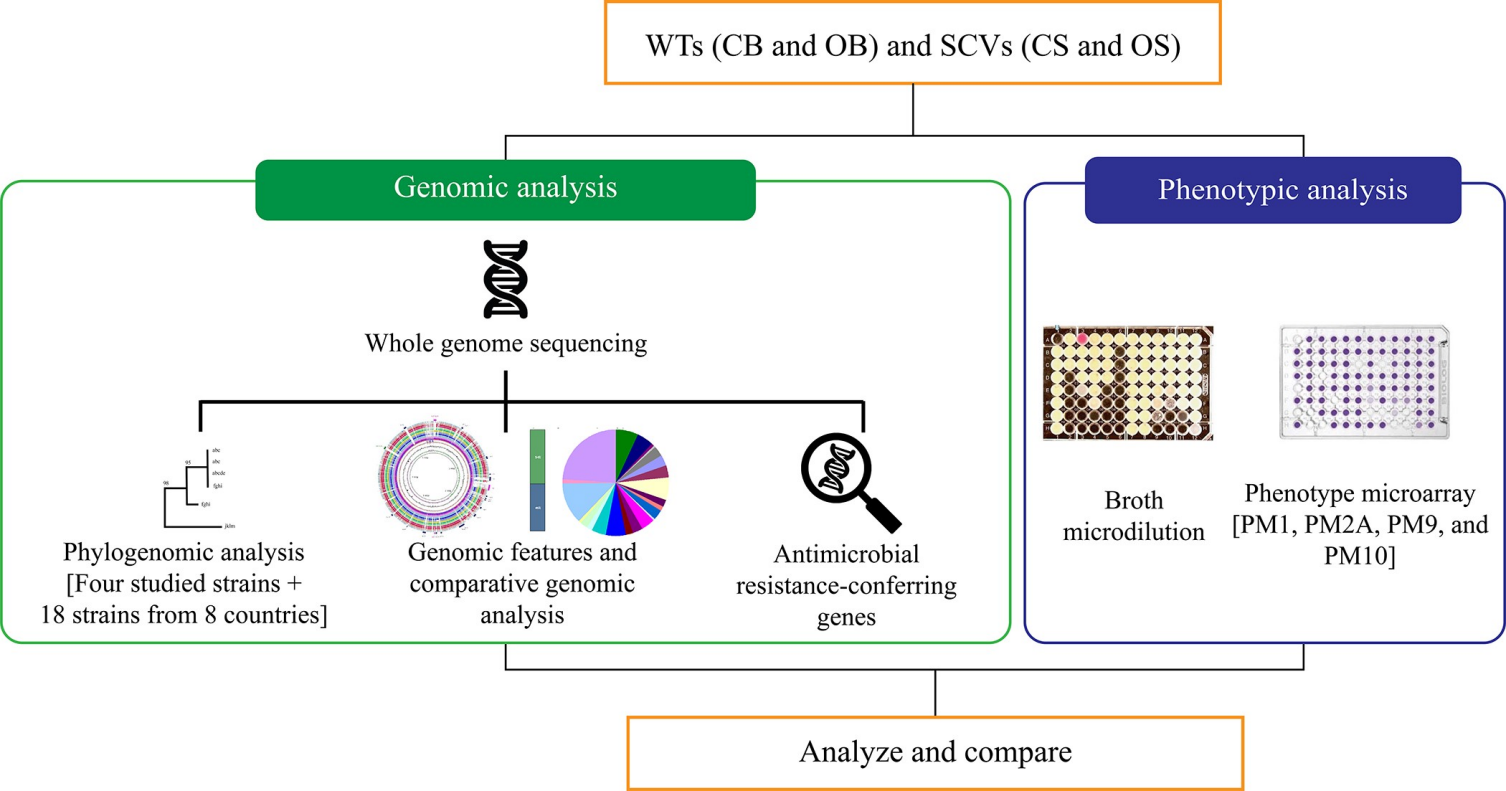
Overview of the study. The WTs and SCVs were subjected to genomic and phenotypic analyses, and the results were analyzed and compared.

In this study, the difference between SCVs and WTs was determined based on genomic and phenotypic characteristics (Fig 1). The SCV and WT isolates were subjected to whole-genome sequencing to determine the genomic contents, including gene functions, subsystems, antimicrobial determinants, and the phylogenomic relatedness with other genomes from different countries. To determine the phenotypic characteristics, the four studied strains were subjected to broth microdilution and phenotype microarray.

### Genome sequencing, assembly and annotation

Genomic DNA was isolated from each strain by using QIAamp DNA Mini Kit (Qiagen, Germany) according to the manufacturer’s instructions. Then, the genome DNAs were sequenced with Illumina MiSeq platform (Illumina Inc., United State of America (USA)). The 250bp sequencing reads were assembled *de novo* using CLC Genomics Workbench 21.0.3 (Qiagen, Germany). The contigs generated were mapped to the reference genome, *B*. *pseudomallei* K96243 (Genbank accession GCA_000959285), and another Malaysia strain, UKMH10 (Genbank accession SAMEA5606430), using Mauve 2.4.0 [17]. The contigs were mapped to UKMH10 as well because UKMH10 was previously subtyped as ST46 [18], which is the same ST as the four studied genomes [16]. Subsequently, the circular genomic maps were constructed separately for chromosome 1 and chromosome 2 using Basic Local Alignment Search Tool (BLAST) ring image generator (BRIG) [19]. The genomic regions of interest were validated using Nucleotide BLAST (BLASTn) and Protein BLAST (BLASTp) (https://blast.ncbi.nlm.nih.gov/Blast.cgi).

The four genome sequences were also submitted and annotated in Rapid Annotation using Subsystem Technology (RAST) (https://rast.nmpdr.org/) server [20–22]. The genomes were subsequently introduced to SEED-viewer environment (https://pubseed.theseed.org/) to map the annotated draft genomes to subsystems (proteins grouped by a relationship in function) and metabolic reconstruction (genes grouped according to a collection of the active variants of subsystems) [20–22].

The genome sequences have been deposited in the National Center for Biotechnology Information (NCBI) GenBank (https://www.ncbi.nlm.nih.gov/genbank/) under the accession number APLM01000000 for CB, APLN01000000 for CS, APLK01000000 for OB, and APLL01000000 for OS. This version described in this paper is the second version.

### Phylogenomic analysis

The four genomes sequenced in this study were compared with 17 publicly available genomes of *B*. *pseudomallei* from different geographical regions (Malaysia, n = 4; Thailand, n = 4; Australia, n = 2; Taiwan, n = 2; Singapore, n = 1; Vietnam, n = 1; Pakistan, n = 1; China, n = 1; USA, n = 1) by using Reference Sequence Alignment-based Phylogenic Builder (RealPhy) Online Tools (https://realphy.unibas.ch/realphy/). *B*. *pseudomallei* K96243 was used as a reference genome while *Burkholderia thailandensis* E264 was used as an outgroup. The genome sequences were retrieved from NCBI GenBank. The country of origin and accession number of each genome is listed in Table 1. The phylogenomic trees were generated separately for chromosome 1 and chromosome 2 and were viewed on FigTree v1.4.4 software (http://tree.bio.ed.ac.uk/software/figtree/).

**Table 1:** List of genomes used for phylogenomic analysis, and country of origin and accession number of the genomes.

Genomes	Country of origin	Accession number
9	Pakistan	SAMN02864945
54	Singapore	SAMEA1483597
406e	Thailand	SAMN03010441
982	Malaysia	SAMN04011951
1026b	Thailand	SAMN02604257
BPHN1	China	SAMN07638450
2011756295	USA	SAMN06007566
HBPUB10303a	Thailand	SAMN02902612
Mahidol-1106a	Thailand	SAMN02866341
MSHR3763	Australia	SAMN04226303
MSHR5858	Australia	SAMN02902606
Pasteur 52237	Vietnam	SAMN02849712
K96243	Thailand	SAMN03075610
UKMH10	Malaysia	SAMEA5606430
UKMPMC2000	Malaysia	SAMEA5606428
UKMR15	Malaysia	SAMEA5606427
vgh16R	Taiwan	SAMN04009759
vgh16W	Taiwan	SAMN04009760

### Antimicrobial resistance-conferring genes identification

AMR-conferring genes were identified by submitting the genome sequences to the Resistance Gene Identifier (RGI) online tool, accessed through the Comprehensive Antibiotic Resistance Database (CARD) (https://card.mcmaster.ca/). RGI predicts AMR genes from the nucleotide data based on homology and SNP models mapped to CARD reference sequences.

### Antimicrobial susceptibility test

Antimicrobial susceptibility testing of the four strains was performed using broth microdilution method in accordance with the Clinical and Laboratory Standards Institute (CLSI) guidelines [23]. *Escherichia coli* ATCC 22952 was used as the quality control organism. The antimicrobial agents tested were amoxicillin-clavulanate, ceftazidime, imipenem, meropenem, and trimethoprim-sulfamethoxazole. Minimum inhibitory concentration (MIC) was determined after 24 hours (for WTs) and 48 hours (for SCVs) of incubation at 37°C.

### Phenotype microarray analysis

The metabolic profile of WTs and SCVs were analyzed using four 96-well Phenotype Microarray (Biolog, Hayward, USA) microplates, which consisted of two microplates of carbon substrates (C-substrates) (PM1 and PM2A), one microplate of various osmolytes concentrations (PM9), and one microplate of various pH conditions (PM10). The C-substrates microplates (PM1 and PM2A) contained a growth control well (A01 well) that contained all nutrients required for cell growth except for C-substrates [24]. The principle of the PM assay relies on the measurement of purple color formation from irreversible redox reaction of the proprietary Biolog tetrazolium dye in response to the production of reduced nicotinamide adenine dinucleotide (NADH) by bacterial respiration. The assay was performed according to the manufacturer’s protocol for Gram-negative bacterium, and the reagents were provided by Biolog (Hayward, USA). Briefly, single colonies of *B*. *pseudomallei* strains were harvested from the third subculture on nutrient agar plates using a sterile cotton swab. For each PM plate, the bacterial cells were suspended into 15 ml of the inoculating fluid [IF-0a GN (1.2 x), dye Mix A (100x) and distilled water] and adjusted to 85% transmittance (T) using a turbidimeter (Biolog Inc.). After the turbidity was adjusted, 100 μL of the prepared bacterial suspensions were added into each well. For PM9 and PM10, a mixture of IF-10a GN (1.2X), dye Mix A (100x), and distilled water was added to the prepared bacterial suspensions before 100uL were distributed into each well. The plate lids were placed and sealed with plastic tape to avoid the wells from drying. The PM plates were incubated in OmniLog® instrument (Biolog, Hayward, USA) at 37°C for 48 hours, where digital images of the plates and colorimetric readings were recorded every 15 minutes at OD_600 nm_. *Klebsiella pneumonia* ATCC 700603 was used as negative control to ensure true positive growth. The identical protocol and incubation conditions for each plate were performed concurrently using this negative control as previously described [25]. The PM assay was conducted in duplicate, on two separate occasions to obtain the true negative and true positive results.

The recorded data were analyzed using OmniLog® PM software (Biolog, Hayward, USA). For each well, a time-course kinetic growth curve was plotted based on the color development of tetrazolium dye. For PM1 and PM2A, the background noise was removed from each well by referencing to the A1 growth control well using the ‘A1 zero’ option. The threshold separating positive growing wells from negative growing wells was set according to the average area under the kinetic growth curve (AUC) and the difference (T_max_ ─T_min_) of the kinetic data obtained in duplicate experiments, as previously described [26]. Positive growth (+) was defined when AUC value of the well is equal to or higher than 1.5 times the AUC value of the negative control, and the T_max_ ─T_min_ value is equal to or higher than OD_600nm_ of 100, which ensured an increasing signal. For PM9 and PM10, only the T_max_ ─T_min_ value was used since these two plates do not have a negative control well. Negative growth (─) was defined when AUC value of the well is 1.5 times lower than the AUC value of the negative control, and/or the T_max_ ─T_min_ value is lower than OD_600nm_ of 100. If the two duplicates had different results, the well was defined as varies growth.

## Results

### Genomic features of *B*. *pseudomallei* draft genomes

The draft genomes of CB, CS, OB, and OS consisted of genome size of 7,154 Kbp, 7,045 Kbp, 7,115 Kbp, and 7,113 Kbp, respectively with a GC content (number of same strand guanine + cytosine sites divided by DNA sequence length) of 68.2%. The genome size and GC content were similar to the reference genome *B*. *pseudomallei* K96243 (7,247 Kbp; 68.2%) [27]. The draft genome of all four strains showed an average N50 value of ~80 Kbp and a high total genome coverage (≥144×). Genome annotation using RAST revealed that CB, CS, OB, and OS contained 7,460, 7,401, 7,388, and 7,360 predicted coding DNA sequences (CDSs), respectively. A summary of the genomic features of these draft genomes are presented in Table 2.

**Table 2:** General genomic features of four *Burkholderia pseudomallei* draft genomes.

Strain	CB	CS	OB	OS
Genome size (bp)	7,154,485	7,045,712	7,115,466	7,113,814
GC content (%)	68.2	68.2	68.2	68.2
N50	79,268	82,228	78,917	81,997
L50	27	28	28	28
Contigs	245	257	245	245
CDS	7,460	7,401	7,388	7,360
Subsystems	374	369	371	372
RNAs	56	56	56	58
Total genome coverage	152×	144×	150×	175×

**Fig 2:**
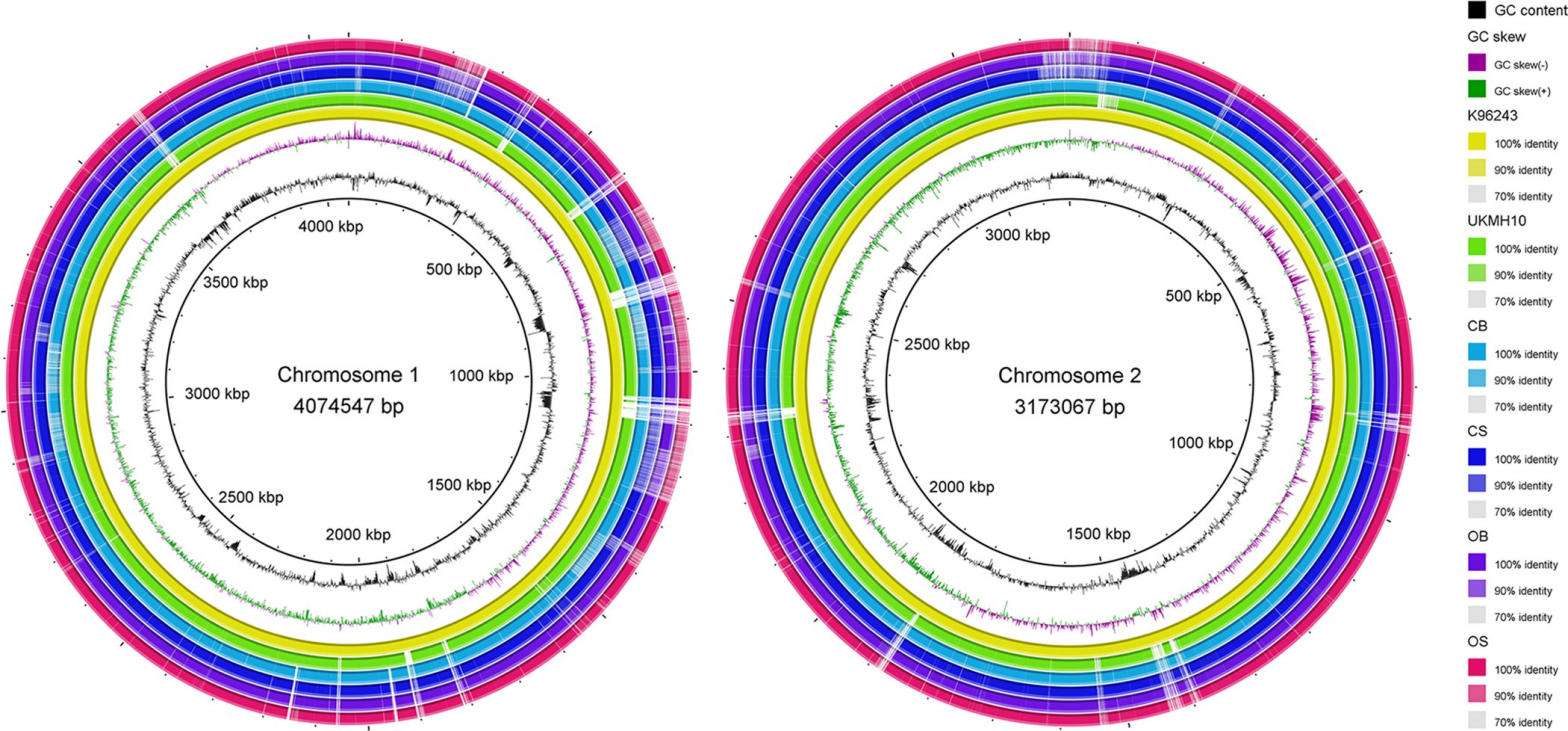
Circular genomic map of chromosome 1 (left) and chromosome 2 (right) in *Burkholderia pseudomallei* K96243, UKMH10, CB, CS, OB, and OS. The total genome size of the reference sequence, K96243, is provided in the center of the rings. The coordinate in scale to K96243 is represented by the innermost ring (black). The black histogram bar represents GC content, whereas the purple-green histogram bar represents GC skew. Colored rings represent orthologous regions of each genome in reference to K96243 genome sequence (yellow ring) and are shown in the following order (innermost to outermost): K96243 (yellow); UKMH10 (green); CB (light blue); CS (dark blue); OB (purple); OS (pink).

The four draft genomes and UKMH10 were compared in reference to *B*. *pseudomallei* K96243 genome. A visual representation of the genomic similarity between these five genomes was depicted by the concentric rings in Fig 2. The missing portions of the rings represented the missing nucleotides in the genomes as compared to *B*. *pseudomallei* K96243 genome. Overall, all five genomes were highly similar to each other as the genomes shared most of the same missing nucleotides regions.

Overall, only 23% of the predicted CDSs in all draft genomes were assigned with subsystems. The most represented subsystem among the four draft genomes was amino acid and derivatives with CDSs ranging from 504 to 517. Other subsystems that were highly presented were associated with carbohydrates, protein metabolism, and cofactors, vitamins, prosthetic groups, pigments. The numbers of annotated genes classified according to subsystems are listed in Table 3.

**Table 3:** A list of subsystems and the number of CDSs corresponding to each subsystem of four *Burkholderia pseudomallei* draft genomes.

Subsystems	Strain			
	CB	CS	OB	OS
**Metabolism**				
Carbohydrates	308	285	291	290
Amino acids and derivatives	506	504	505	517
Fatty acids, lipids, and isoprenoids	125	119	117	118
Cofactors, vitamins, prosthetic groups, pigments	214	219	210	211
Protein metabolism	223	222	222	223
Sulfur metabolism	29	29	30	32
Iron acquisition and metabolism	47	45	48	49
Potassium metabolism	8	8	8	8
RNA metabolism	58	58	58	58
Secondary metabolism	8	9	8	9
DNA metabolism	67	68	68	71
Nitrogen metabolism	65	64	67	65
Metabolism of aromatic compounds	83	82	83	84
Nucleosides and nucleotides	92	94	94	93
Phosphorus metabolism	31	31	31	31
**Cellular processes**				
Cell wall and capsule	56	58	59	58
Motility and chemotaxis	60	60	18	18
Regulation and cell signalling	35	32	34	35
**Environments Information Processing**				
Membrane transport	164	164	162	163
**Virulence**				
Virulence, disease, and defence	65	67	65	63
Phages, prophages, transposable elements, plasmids	13	4	9	10
Stress response	95	94	96	95
**Others**				
Dormancy and sporulation	1	1	1	1
Respiration	153	151	149	153
Miscellaneous	49	49	49	50
**Total in subsystems (%)**	**23**	**23**	**23**	**23**
Total	1715	1696	1678	1686
Non-hypothetical	1641	1623	1606	1614
Hypothetical	74	73	72	72
**Total not in subsystems (%)**	**77**	**77**	**77**	**77**
Total	5745	5705	5710	5674
Non-hypothetical	2909	2913	2957	2941
Hypothetical	2836	2792	2753	2733

### Phylogenetic analysis

**Fig 3:**
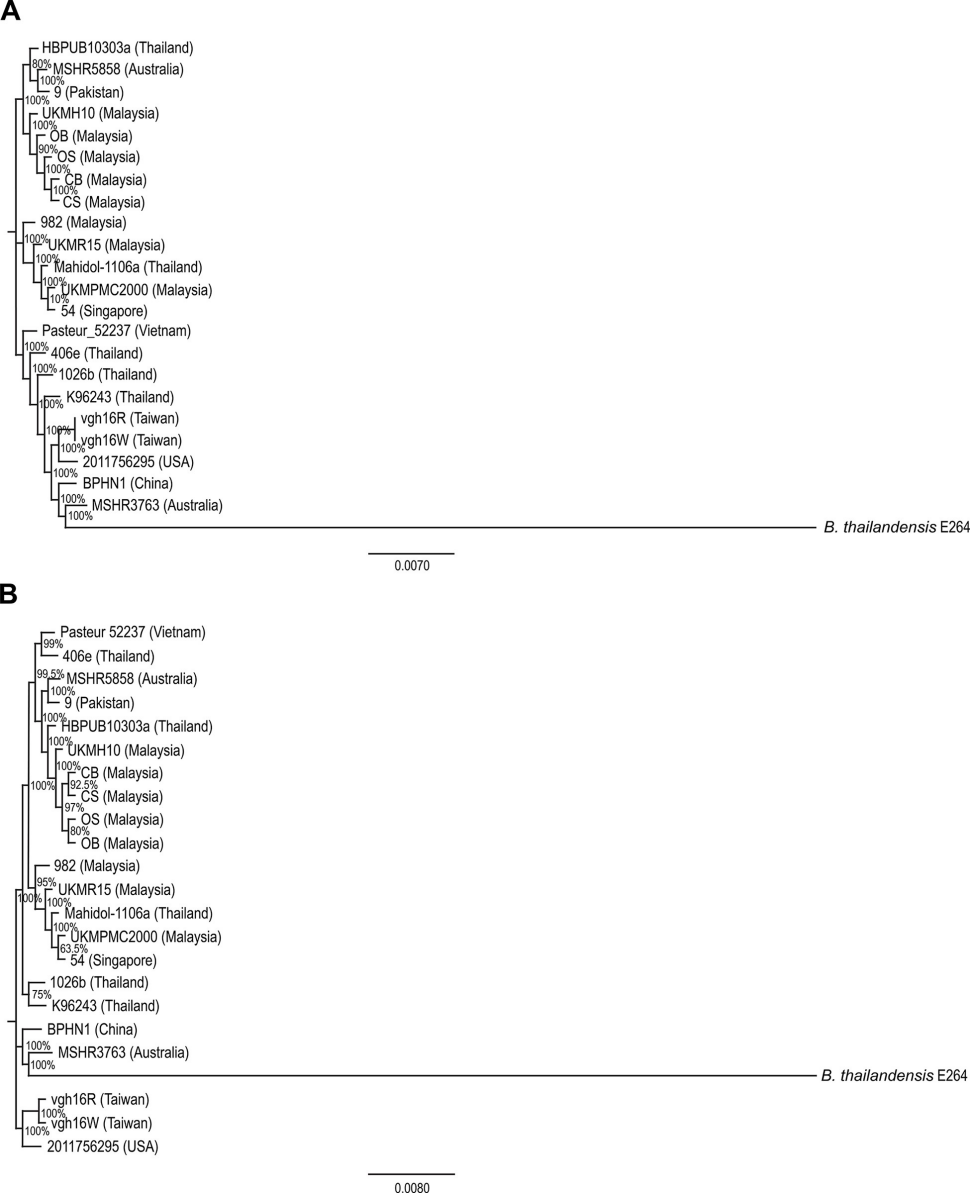
The phylogenomic tree inferred based on chromosome 1 (A) and chromosome 2 (B) of the representative *Burkholderia pseudomallei* strains. The core genome SNP-based alignment was generated by REALPHY server using *B*. *pseudomallei* strain K96243 (chromosome 1 (A) and chromosome 2 (B)) as the reference genome. The unrooted phylogenomic tree was inferred by approximately Maximum Likelihood (ML) method using Generalized Time-Reversible (GTR) model with gamma distribution of rates. 100 bootstrap replicates were used to infer the ML tree and the support value is indicated as a percentage at each node. The *B*. *thailandensis* strain E264 represents a phylogenetic outgroup.

The four draft genomes from this study and 19 publicly available genomes were compared using single nucleotide polymorphism (SNP)-based phylogenetic analysis based on chromosome 1 (Fig 3A) and chromosome 2 (Fig 3B). Overall, the phylogenetic tree generated revealed that the genomes from Southeast Asia (Malaysia, Thailand, Singapore, and Vietnam) were clustered together, with the inclusion of two genomes from Australia (MSHR5858) and Pakistan (9). Meanwhile, the other genomes from China, Taiwan, USA, and Australia formed a separate cluster. The four genomes in this study formed a tight cluster (node-supporting value >90%) together with another strain from Malaysia, UKMH10. These five genomes also shared a node with a cluster consisted of HBPUB1303a (Thailand), 9 (Pakistan) and MSHR5858 (Australia).

### Antimicrobial resistance-conferring genes

The AMR-conferring genes identified in each genome are compiled in Table 4. All four strains carried *amr*B, *omp*38, OXA-57 and *ade*F genes. OXA-57 encodes OXA beta-lactamase that inactivates antibiotic binding site. The *omp*38 gene encodes a bacterial porin that has reduced permeability towards beta-lactams. Both *ade*F and *amr*B encode resistance-nodulation-cell division (RND) antibiotic efflux pump.

**Table 4:** Predicted antimicrobial resistance genes in *Burkholderia pseudomallei* strains.

Predicted gene	Gene family	Gene identity (%)			
		CB	CS	OB	OS
***amr*B**	Resistance-nodulation-cell division (RND) antibiotic efflux pump	100	100	100	100
***omp*38**	Bacterial porin	100	100	100	100
**OXA-57**	OXA beta lactamase	99.63	99.63	99.63	99.63
***ade*F**	Resistance-nodulation-cell division (RND) antibiotic efflux pump	79.49	79.49	79.49	79.49

### Antimicrobial susceptibility testing

Antimicrobial susceptibility test revealed that all strains were susceptible to amoxicillin-clavulanate, ceftazidime, imipenem, meropenem and trimethoprim-sulfamethoxazole (Table 5).

**Table 5:** Antimicrobial susceptibility profiles of *Burkholderia pseudomallei* strains.

Antimicrobial agents	CLSI susceptibility breakpoint (μg/ml)^a^			Susceptibility of *B*. *pseudomallei* strains (MIC, μg/ml)^a^			
	S	I	R	CB	CS	OB	OS
Amoxicillin-clavulanate	≤8/4	16/8	≥32/16	4 (S)	4 (S)	4 (S)	4 (S)
Ceftazidime	≤8	16	≥32	4 (S)	1 (S)	2 (S)	4 (S)
Imipenem	≤4	8	≥16	0.5 (S)	0.5 (S)	0.5 (S)	0.5 (S)
Meropenem^b^	≤4	8	≥16	2 (S)	2 (S)	2 (S)	1 (S)
Trimethoprim-sulfamethoxazole	≤2/38	-	≥4/76	1 (S)	0.5 (S)	0.5 (S)	1 (S)

^a^ CLSI, Clinical and Laboratory Standards Institute; MIC, minimum inhibitory concentration; S, susceptible; I, intermediate; R, resistant.

^b^ MIC breakpoint value is not available for meropenem, thus CLSI breakpoint value for imipenem is used [28].

### Carbon substrates utilization

Based on the PM1 and PM2 carbon utilization, the core (100% utilization of the strains) and differential carbon sources (utilized by at least one of the strains) utilized by the strains were identified (Table 6). Among the 190 C-substrates from both plates (95 C-substrates in each PM plate), all strains utilized 60 C-substrates, particularly carboxylic acid compounds (n = 27 out of 60; 45%). OB and OS utilized 13 C-substrates more than CB and CS. No significant difference was observed between the WTs (CB-OB) and SCVs (CS-OS) in the utilized C-substrates.

**Table 6:** Summary of phenotype microarray results.

Category	Carbon source	Strains			
		CB	CS	OB	OS
**Amino acid**	L-Proline	+	+	+	+
	D-Alanine	+	+	+	+
	L-Glutamic acid	+	+	+	+
	L-Asparagine	+	+	+	+
	L-Glutamine	+	+	+	+
	L-Serine	+	+	+	+
	L-Alanine	+	+	+	+
	Ala-Glycine	+	+	+	+
	L-Histidine	+	+	+	+
	Hydroxy-L-Proline	+	+	+	+
	L-Phenylalanine	+	+	+	+
	L-Aspartic acid	–	–	+	+
	D-Serine	–	+	+	+
	L-Threonine	–	–	+	+
	L-Arginine	–	+	+	+
	L-Isoleucine	–	–	+	+
	L-Leucine	–	–	+	+
	L-Pyroglutamic acid	–	–	+	+
	L-Valine	–	–	+	+
**Carbohydrate**	N-Acetyl-D-Glucosamine	+	+	+	+
	D-Galactose	+	+	+	+
	D-Trehalose	+	+	+	+
	Dulcitol	+	+	+	+
	D-Sorbitol	+	+	+	+
	Glycerol	+	+	+	+
	L-Fucose	+	+	+	+
	D,L-a-Glycerol Phosphate	+	+	+	+
	D-Mannitol	+	+	+	+
	D-Glucose-6-Phosphate	+	+	+	+
	D-Fructose	+	+	+	+
	a-D-Glucose	+	+	+	+
	D-Fructose-6-Phosphate	+	+	+	+
	m-Inositol	+	+	+	+
	N-Acetyl-D-Galactosamine	+	+	+	+
	D-Arabinose	+	+	+	+
	D-Arabitol	+	+	+	+
	D-Mannose	–	+	+	+
	D-Ribose	+	–	+	+
	D-Glucose-1-Phosphate	–	–	+	–
	i-Erythritol	–	–	+	+
**Carboxylic acid**	Succinic acid	+	+	+	+
	D-Gluconic acid	+	+	+	+
	L-Lactic acid	+	+	+	+
	Formic acid	+	+	+	+
	D,L-Malic acid	+	+	+	+
	Acetic acid	+	+	+	+
	D-Glucosaminic acid	+	+	+	+
	a-Ketoglutaric acid	+	+	+	+
	a-Ketobutyric acid	+	+	+	+
	a-Hydroxyglutaric acid-g-Lactone	+	+	+	+
	a-Hydroxybutyric acid	+	+	+	+
	Fumaric acid	+	+	+	+
	Bromosuccinic acid	+	+	+	+
	Propionic acid	+	+	+	+
	L-Malic acid	+	+	+	+
	p-Hydroxyphenyl Acetic acid	+	+	+	+
	Pyruvic acid	+	+	+	+
	g-Amino-N-Butyric acid	+	+	+	+
	Butyric acid	+	+	+	+
	Caproic acid	+	+	+	+
	Dihydroxyfumaric acid	+	+	+	+
	4-Hydroxybenzoic acid	+	+	+	+
	b-Hydroxybutyric acid	+	+	+	+
	Malonic acid	+	+	+	+
	Quinic acid	+	+	+	+
	Sebacic acid	+	+	+	+
	Sorbic acid	+	+	+	+
	D-Galactonic acid-g-Lactone	–	+	+	+
	Citric acid	–	–	+	+
	Mono-Methylsuccinate	–	–	+	+
	g-Hydroxybutyric acid	–	–	–	+
	Succinamic acid	–	–	+	+
	D,L-Carnitine	–	+	–	–
**Fatty acid**	Tween 20	+	+	+	+
	Tween 40	+	+	+	+
	Tween 80	+	+	+	+
**Amine**	D,L-Octopamine	+	+	+	+
	Phenylethylamine	–	–	+	+
	Putrescine	–	–	+	+
**Amide**	L-Alaninamide	–	+	+	+
**Alcohol**	2-Aminoethanol	–	–	+	+
**Ester**	Methylpyruvate	+	+	+	+
**Polymer**	Gelatin	–	+	+	+

Notes: ‘+’, positive growth; ‘─’, negative growth.

**Fig 4:**
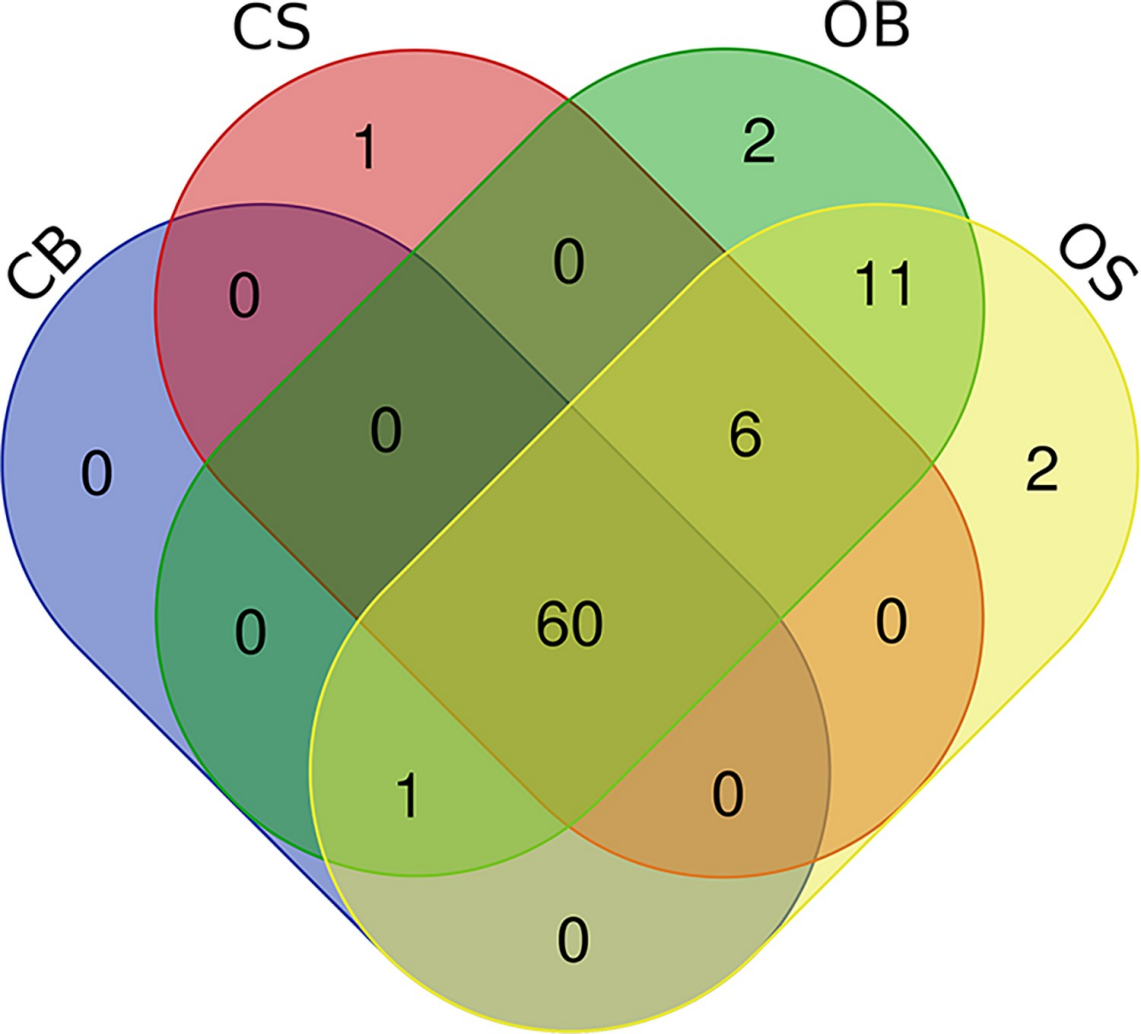
Venn diagram showing the number of core, differential and strain-specific carbon utilization of *Burkholderia pseudomallei* strains. The results obtained from the panels PM1 and PM2A are compiled in this schematic diagram.

Both OB and OS utilized a total of 81 C-substrates, but two of the C-substrates were different between the two strains. D-glucose-1-phosphate and L-Valine were uniquely utilized by OB while g-hydroxybutyric acid and succinamic acid were uniquely utilized by OS. Also, D,L-carnitine was only metabolized by CS. A Venn diagram was constructed to illustrate the core and differential C-substrates utilized by the *B*. *pseudomallei* strains (Fig 4).

### Osmolytes sensitivity

Based on the osmolarity responses (PM9) (Table 7), all the four strains were able to grow in 1% NaCl, but sensitive (no growth) on a higher concentration of salt. We also observed that all strains were able to grow in sodium sulfate (2–5%), ethylene glycol (5–20%), sodium lactate (1–4%), sodium phosphate pH 7 (20-200mM), ammonium sulfate pH 8 (10–100 mM), and sodium nitrate (10–100 mM).

**Table 7:** Phenotype microarray results for PM9 and PM10.

Well	PM conditions	Remarks	Strains			
			CB	CS	OB	OS
**PM9**	**Osmolarity sensitivity**					
**A01**	1% NaCl	osmotic sensitivity, NaCl	+	+	+	+
**D05**	2% Sodium Sulfate	osmotic sensitivity, Na_2_SO_4_	+	+	+	+
**D06**	3% Sodium Sulfate	osmotic sensitivity, Na_2_SO_4_	+	+	+	+
**D07**	4% Sodium Sulfate	osmotic sensitivity, Na_2_SO_4_	+	+	+	+
**D08**	5% Sodium Sulfate	osmotic sensitivity, Na_2_SO_4_	+	+	+	+
**D09**	5% Ethylene Glycol	osmotic sensitivity, ethylene glycol	+	+	+	+
**D10**	10% Ethylene Glycol	osmotic sensitivity, ethylene glycol	+	+	+	+
**D11**	15% Ethylene Glycol	osmotic sensitivity, ethylene glycol	+	+	+	+
**D12**	20% Ethylene Glycol	osmotic sensitivity, ethylene glycol	+	+	+	+
**E01**	1% Sodium Formate	osmotic sensitivity, sodium formate	+	+	+	+
**E02**	2% Sodium Formate	osmotic sensitivity, sodium formate	+	–	–	V
**E07**	2% Urea	osmotic sensitivity, urea	+	+	+	+
**E08**	3% Urea	osmotic sensitivity, urea	+	+	V	+
**F01**	1% Sodium Lactate	osmotic sensitivity, sodium lactate	+	+	+	+
**F02**	2% Sodium Lactate	osmotic sensitivity, sodium lactate	+	+	+	+
**F03**	3% Sodium Lactate	osmotic sensitivity, sodium lactate	+	+	+	+
**F04**	4% Sodium Lactate	osmotic sensitivity, sodium lactate	+	+	+	+
**G01**	20mM Sodium Phosphate pH 7	osmotic sensitivity, sodium phosphate	+	+	+	+
**G02**	50mM Sodium Phosphate pH 7	osmotic sensitivity, sodium phosphate	+	+	+	+
**G03**	100mM Sodium Phosphate pH 7	osmotic sensitivity, sodium phosphate	+	+	+	+
**G04**	200mM Sodium Phosphate pH 7	osmotic sensitivity, sodium phosphate	+	+	+	+
**G05**	20mM Sodium Benzoate pH 5.2	toxicity, benzoate	+	+	+	+
**G09**	10mM Ammonium Sulfate pH 8	toxicity, ammonia	+	+	+	+
**G10**	20mM Ammonium Sulfate pH 8	toxicity, ammonia	+	+	+	+
**G11**	50mM Ammonium Sulfate pH 8	toxicity, ammonia	+	+	+	+
**G12**	100mM Ammonium Sulfate pH 8	toxicity, ammonia	+	+	+	+
**H01**	10mM Sodium Nitrate	toxicity, nitrate	+	+	+	+
**H02**	20mM Sodium Nitrate	toxicity, nitrate	+	+	+	+
**H03**	40mM Sodium Nitrate	toxicity, nitrate	+	+	+	+
**H04**	60mM Sodium Nitrate	toxicity, nitrate	+	+	+	+
**H05**	80mM Sodium Nitrate	toxicity, nitrate	+	+	+	+
**H06**	100mM Sodium Nitrate	toxicity, nitrate	+	+	+	+
**H07**	10mM Sodium Nitrite	toxicity, nitrite	+	+	+	+
**H08**	20mM Sodium Nitrite	toxicity, nitrite	+	+	+	+
**H09**	40mM Sodium Nitrite	toxicity, nitrite	+	+	+	+
**H10**	60mM Sodium Nitrite	toxicity, nitrite	+	+	+	+
**H11**	80mM Sodium Nitrite	toxicity, nitrite	+	+	+	–
**PM 10**	**pH conditions**					
**A03**	pH 4.5	pH, growth at 4.5	+	+	+	V
**A04**	pH 5	pH, growth at 5	+	+	+	+
**A05**	pH 5.5	pH, growth at 5.5	+	+	+	+
**A06**	pH 6	pH, growth at 6	+	+	+	+
**A07**	pH 7	pH, growth at 7	+	+	+	+
**A08**	pH 8	pH, growth at 8	+	+	+	+
**A09**	pH 8.5	pH, growth at 8.5	+	+	+	+
**A10**	pH 9	pH, growth at 9	+	–	+	–
**B01**	pH 4.5	pH, decarboxylase control	+	+	+	V
**B02**	pH 4.5 + L-Alanine	pH, decarboxylase	+	+	+	V
**B03**	pH 4.5 + L-Arginine	pH, decarboxylase	+	+	+	–
**B04**	pH 4.5 + L-Asparagine	pH, decarboxylase	–	+	+	–
**B05**	pH 4.5 + L-Aspartic acid	pH, decarboxylase	+	V	+	–
**B06**	pH 4.5 + L-Glutamic acid	pH, decarboxylase	–	–	+	–
**B07**	pH 4.5 + L-Glutamine	pH, decarboxylase	–	–	+	+
**B08**	pH 4.5 + Glycine	pH, decarboxylase	+	V	+	–
**B09**	pH 4.5 + L-Histidine	pH, decarboxylase	+	V	+	–
**B12**	pH 4.5 + L-Lysine	pH, decarboxylase	+	V	+	–
**C01**	pH 4.5 + L-Methionine	pH, decarboxylase	–	+	+	V
**C02**	pH 4.5 + L-Phenylalanine	pH, decarboxylase	–	V	+	–
**C03**	pH 4.5 + L-Proline	pH, decarboxylase	+	+	+	–
**C04**	pH 4.5 + L-Serine	pH, decarboxylase	+	+	+	+
**C05**	pH 4.5 + L-Threonine	pH, decarboxylase	+	+	+	+
**C07**	pH 4.5 + L-Tyrosine	pH, decarboxylase	+	+	+	V
**C08**	pH 4.5 + L-Valine	pH, decarboxylase	+	+	+	V
**C09**	pH 4.5 + Hydroxy-L-Proline	pH, decarboxylase	+	+	+	V
**C10**	pH 4.5 + L-Ornithine	pH, decarboxylase	+	+	+	–
**C11**	pH 4.5 + L-Homoarginine	pH, decarboxylase	+	+	+	–
**D03**	pH 4.5 + L-Norvaline	pH, decarboxylase	+	+	+	+
**D04**	pH 4.5 + a-Amino-N-Butyric acid	pH, decarboxylase	+	+	+	V
**D06**	pH 4.5 + L-Cysteic acid	pH, decarboxylase	+	+	+	V
**D07**	pH 4.5 + D-Lysine	pH, decarboxylase	+	+	+	+
**D08**	pH 4.5 + 5-Hydroxy-L-Lysine	pH, decarboxylase	+	+	+	V
**D10**	pH 4.5 + D,L-Diamino-a,e-Pimelic acid	pH, decarboxylase	–	–	V	V
**D11**	pH 4.5 + Trimethylamine-N-Oxide	pH, decarboxylase	V	V	+	–
**D12**	pH 4.5 + Urea	pH, decarboxylase	+	+	+	V
**H01**	X-Caprylate	caprylate esterase	+	+	+	+
**H02**	X-a-D-Glucoside	a-D-glucosidase	+	+	+	+
**H03**	X-b-D-Glucoside	b-D-glucosidase	+	+	+	+
**H04**	X-a-D-Galactoside	a-D-galactosidase	+	+	+	+
**H05**	X-b-D-Galactoside	b-D-galactosidase	+	+	+	+
**H06**	X-a-D-Glucuronide	a-D-glucuronidase	+	+	+	+
**H07**	X-b-D-Glucuronide	b-D-glucuronidase	+	+	+	+
**H08**	X-b-D-Glucosaminide	b-D-glucosaminidase	+	+	+	+
**H09**	X-b-D-Galactosaminide	b-D-galactosaminidase	+	+	+	+
**H10**	X-a-D-Mannoside	a-D-mannosidase	+	+	+	+
**H11**	X-PO4	aryl phosphatase	+	+	+	+
**H12**	X-SO4	aryl sulfatase	+	+	+	+

Notes: ‘+’, positive growth; ‘─’, negative growth; V, varies growth between replicates.

### pH conditions

As for the pH sensitivity (PM10) (Table 7), the strains were able to grow from pH 5 to pH 8.5. However, only the WT strains (CB and OB) were able to grow in pH 9. Both SCVs showed variable responses for pH 4.5 with certain decarboxylase in all three replicates despite our effort to standardize the protocol.

The growth patterns of the negative control strain on PM1, PM2A, PM9 and PM10 microplates were significantly different compared to the studied strains. No significant difference was observed in the two runs conducted separately. Hence, the true positive growth was determined.

## Discussion

In this study, genotypic and phenotypic characteristics of two SCVs (CS and OS) and their corresponding WTs (CB and OB) were determined. SNP-based phylogenetic analysis revealed that the four draft genomes were more closely related to UKMH10 (Malaysia) compared to the reference genome, K96243 (Thailand). The finding is supported by MLST results as K96243 was assigned as ST10 [29] while the four strains and UKMH10 were assigned as ST46 [16,18]. According to the allelic profile of these two STs, the genomes only shared three identical alleles at loci of *glt*B, *lep*A and *lip*A. The difference in genome contents of K96243 compared to the five genomes of ST46 was also demonstrated in the circular genomic map, whereby a number of missing nucleotide regions were identified. However, the four draft genomes did not show any gap of approximately 20kb along chromosome 2 as reported in UKMH10 [18]. In addition to MLST results, the four draft genomes were previously characterized by PFGE, which revealed that OB and OS only shared 67% similarity in pulsotypes while CB and CS shared 95% similarity in pulsotypes [16]. The discrepancy between MLST and PFGE results has now been resolved as the close relationship of OB and OS was supported by a high node-supporting value (90% for chromosome 1; 80% for chromosome 2) based on phylogenetic tree analysis. Hence, the finding in this study confirmed the close relationship (clonal) between the SCVs and their respective WTs.

The four draft genomes were found to be closely related to the other genomes from Southeast Asia and two genomes from other regions; MSHR5858 (Australia) and 9 (Pakistan). Previous study has suggested that MSHR5858, assigned as ST562, was from an Asian origin based on both eBURST analysis and whole-genome SNP-based phylogenetics tree [30]. As for the genome from Pakistan, there has been lack of information about the true origin of the genome [31]. However, previous study showed that a genome from Pakistan was clustered together with genomes from Bangladesh, Myanmar, Laos, Thailand, China, Malaysia and Indonesia based on SNP-based phylogeny analysis [32].

In general, SCVs population has been reported to be more resistant towards antimicrobial agents compared to their parental strain [8,33,34]. Thus, the susceptibility of *B*. *pseudomallei* SCV and WT strains in this study were tested with five antimicrobial agents (amoxicillin-clavulanate, ceftazidime, imipenem, meropenem and trimethoprim-sulfamethoxazole) which are commonly used for meliodosis treatment [35,36]. However, all of the strains were susceptible to these antimicrobial agents. The results were supported by AMR-conferring genes found in the four draft genomes. Among the genes identified, OXA-57 was associated with resistance to cephalosporin and penam through inactivation of the antibiotic binding site [37] while *ade*F was associated with resistance to tetracycline and fluoroquinolones [38]. These antimicrobial groups were not used in this study since *B*. *pseudomallei* is inherently resistant to these agents [39]. The findings suggested that the lack of AMR-conferring genes associated with the five antimicrobial agents used may have resulted in susceptibility towards the antimicrobial agents which are important in melioidosis treatment.

*B*. *pseudomallei* is an intracellular pathogen and thus, the survival of this bacterium inside host cells depends on their acquisition of carbon sources from the host environment [40,41]. Based on phenotype microarray results, all *B*. *pseudomallei* strains were able to utilize α-D-glucose, D-glucose-6-phosphate, D-fructose, and D-arabitol, which are among the important C-substrates for glycolysis. Meanwhile, both SCVs and WTs utilized L-proline, L-histidine, L-glutamine, L-glutamic acid, which are involved in the synthesis of intermediate for tricarboxylic acids (TCA) cycle (i.e., α-ketoglutarate). Metabolic pathways inferred from SEED showed that the four *B*. *pseudomallei* strains also harboured genes encoded for glycolysis, pentose phosphate cycle and Entner-Doudoroff pathways. The findings were consistent with the genomic analysis of other *B*. *pseudomallei* strains (K96243, MSHR668 and 1106a) which also demonstrated the involvement of *B*. *pseudomallei* in these three metabolic pathways [42].

Previous study by Eisenreich *et al*. showed that intracellular bacteria prefer substrates such as glycerol over glucose during infection [40]. In this study, all *B*. *pseudomallei* strains were able to utilize glycerol. Glycerol feeds in the middle of the glycolysis/gluconeogenesis pathways with the production of glyceraldehyde 3-phosphate (KEGG database). Moreover, the strains were also able to utilize tween 20, tween 40 and tween 60 which are fatty acids. Previous study on *Mycobacterium tuberculosis* revealed that the bacteria metabolized glycerol and fatty acids as carbon sources in the macrophage environment [43].

When comparing SCVs with their parental WTs based on the phenotypic profile, the only difference observed was the ability to grow in pH 9. In our study, both morphology variants were able to grow in the pH range of 4.5 to 8.5. This is consistent with a previous study that reported the pH range of *B*. *pseudomallei* at 4.5–7 [44]. However, the WT strains in this study were able to grow up to pH 9. Similarly, in a rare case reported by Finkelstein *et al*. [45], *B*. *pseudomallei* has been isolated from water sources with pH value ranging from pH 2 to pH 9 [44]. In general, the survival of *B*. *pseudomallei* at acidic condition is relatively low since pH 4 or lower is considered to be bactericidal. On the contrary, a previous study reported that the ability of *B*. *pseudomallei* to survive in acidic condition may be attributed to over-expression of components of the arginine deiminase system (i.e. arginine deiminase and carbamate kinase), however, this does not provide any advantage to bacterial intracellular survival or replication [46].

Rather than differentiating WTs and SCVs based on their metabolic phenotypes, significant differences were observed between the two pair of strains, whereby OB and OS were found to utilize more C-substrates (n = 81) than CB and CS (n = 61 and n = 67, respectively). The ability to utilize more C-substrates suggested that OB and OS may survive and adapt better under stressed environments. Although all four strains were found to be genotypically similar, the difference in nutrient utilization could be due to epigenetic factors and/or gene expression regulation rather than genomic differences [47]. It was discussed previously that within different SCV populations, the commonality between phenotypic and genotypic changes was not found [3]. Thus, the finding suggested that carbon utilization may be strain-specific rather than morphotype-specific.

The limitation of this study was unavailability of the patients’ clinical history such as antibiotics administered, severity of the infection (bacteremia, septic shock, or death) and clinical manifestations. Availability of such information may provide a better insight into the pathogenesis or virulence of the bacteria in human host.

Based on the data obtained from phenotypic microarray, each pair of colonial variants (CB-CS and OB-OS) utilized the carbon more similarly for growth while only WTs (CB and OB) could survive at pH 9. However, based on SNP-based phylogenetic analysis, all the four studied strains were closely related with the other 18 *B*. *pseudomallei* strains from 8 different countries.
